# Can risk scoring systems (R.E.N.A.L, PADUA and (MC)2 scoring systems) and related risk factors predict complications of renal ablation? a meta-analysis

**DOI:** 10.3389/fonc.2026.1753281

**Published:** 2026-08-21

**Authors:** Zhiqiang Zeng, Peng Ji, Yang He, Yubo Zhou, Tao Li, Lunhong Zou, Tao Zhou, Huan Zhao, Xionglin Hu, Wangbing Chen, Wubin Chen

**Affiliations:** 1 Santai People‘s Hospital (Santai Hospital Affiliated to North Sichuan Medical College), Mianyang, Sichuan, China; 2 North Sichuan Medical College (University), Nanchong, Sichuan, China

**Keywords:** (MC)2 scoring system, meta-analysis, PADUA scoring system, R.E.N.A.L. scoring system, renal ablation

## Abstract

**Objective:**

To evaluate the predictive value of the R.E.N.A.L., PADUA, and (MC)^2^ scoring systems for complications after renal tumor ablation.

**Method:**

This systematic review and meta-analysis was reported in accordance with PRISMA and registered in PROSPERO. PubMed, Embase, the Cochrane Library, and Web of Science were searched from inception to May 1, 2025. The primary outcomes were overall, major, and minor complications after renal ablation in relation to the R.E.N.A.L., PADUA, or (MC)^2^ scoring systems.

**Results:**

A total of 25 retrospective studies involving 5,583 patients were included. Higher R.E.N.A.L., PADUA, and (MC)^2^ score categories were associated with higher odds of major complications after renal ablation. Exploratory analyses suggested that larger tumor diameter, closer proximity to the collecting system, central tumor location, complicated diabetes mellitus, and history of myocardial infarction may be associated with increased odds of complications, although several of these analyses were based on only a small number of studies.

**Conclusions:**

The R.E.N.A.L., PADUA, and (MC)^2^ scoring systems may help stratify complication risk after renal ablation, particularly for major complications. Findings regarding individual risk factors, especially complicated diabetes mellitus, history of myocardial infarction, and central tumor location, should be considered hypothesis-generating and require validation in larger prospective studies.

**Systematic Review Registration:**

https://www.crd.york.ac.uk/prospero/, identifier CRD42024551193.

## 1 Introduction

Renal ablation, including radiofrequency ablation, microwave ablation, and cryoablation, has become an important minimally invasive treatment option for selected patients with small renal masses, particularly those with T1a tumors or increased surgical risk (1–3). Although renal ablation is generally considered safe, procedure-related complications may still occur, especially in patients with anatomically complex tumors or relevant comorbidities (4, 5). Accurate preprocedural prediction of complications is clinically important because it may support patient counselling, treatment selection, procedural planning, use of adjunctive protective techniques, and postoperative monitoring (6, 7).

Several nephrometry or risk scoring systems have been used to quantify tumor complexity before renal tumor treatment (8). The R.E.N.A.L. and PADUA scores were originally developed to standardize the anatomical assessment of renal tumors, mainly in the setting of nephron-sparing surgery, rather than specifically to predict ablation-related complications. These systems assess features such as tumor size, exophytic or endophytic growth pattern, tumor depth, anatomical location, and proximity to the collecting system or renal sinus (9). Because these anatomical characteristics may also influence probe placement, ablation margins, energy delivery, and the risk of injury to adjacent renal structures, the predictive value of R.E.N.A.L. and PADUA scores for renal ablation complications deserves further evaluation (10). In contrast, the (MC)^2^ score was developed specifically for patients undergoing renal ablation and incorporates both tumor-related and patient-related factors, including maximum tumor diameter, central tumor location, history of myocardial infarction, and complicated diabetes mellitus (11, 12).

Previous studies have evaluated the association between nephrometry or ablation-specific risk scores and complications after renal ablation, but the available evidence remains fragmented (13). Most studies were retrospective, and they differed in ablation modality, procedural approach, and the scoring systems evaluated (14). In addition, many studies focused on a single scoring system or selected risk factors, making it difficult to determine the overall clinical utility of these tools (15). To our knowledge, no previous meta-analysis has simultaneously synthesized the available evidence on the R.E.N.A.L., PADUA, and (MC)^2^ scoring systems for predicting complications after renal ablation while also evaluating related risk factors. Therefore, we conducted this systematic review and meta-analysis to assess the predictive value of these scoring systems and to summarize clinically relevant risk factors for renal ablation complications.

## 2 Methods

### 2.1 Literature search

This systematic review and meta-analysis was reported in accordance with the PRISMA statement (16) and was prospectively registered in PROSPERO (registration number: CRD42024551193).

A comprehensive literature search was performed in PubMed, Embase, the Cochrane Library, and Web of Science from database inception to May 1, 2025. The search strategy combined terms related to renal tumors, renal ablation, nephrometry or risk scoring systems, and complications. The main search terms included “renal tumor,” “kidney tumor,” “renal mass,” “renal cell carcinoma,” “ablation,” “cryoablation,” “radiofrequency ablation,” “microwave ablation,” “R.E.N.A.L.,” “RENAL nephrometry,” “PADUA,” “(MC)^2^,” “MC2,” “risk score,” “scoring system,” “complication,” and “adverse event.” No language restrictions were applied during the database search. The full search strategy for each database is provided in **Supplementary Table 1**.

Two reviewers independently performed the literature search and screened the titles and abstracts. The full texts of potentially eligible studies were then reviewed independently by the same reviewers. Disagreements were resolved by discussion, and unresolved disagreements were adjudicated by a third reviewer. In addition, the reference lists of eligible studies and relevant reviews were manually searched to identify additional studies.

### 2.2 Eligibility criteria

Studies were included if they met all of the following criteria: (1) Population: human patients with renal tumors or small renal masses who underwent renal ablation; (2) Intervention/procedure: renal ablation performed using radiofrequency ablation, microwave ablation, cryoablation, or other thermal ablation techniques, regardless of whether the procedure was performed percutaneously, laparoscopically, or by another approach; (3) Exposure: patients were classified according to at least one of the following scoring systems: the R.E.N.A.L. nephrometry score, the PADUA score, or the (MC)^2^ score; alternatively, studies were eligible if they reported prespecified related risk factors or score components in relation to complications; (4) Outcome: the study reported post-ablation complications, including overall complications, major complications, minor complications, or sufficient data to calculate these outcomes; (5) Study type: observational cohort studies, case-control studies, or clinical studies with extractable comparative data. In this review, “relevant scoring systems” referred specifically to the R.E.N.A.L., PADUA, and (MC)^2^ scoring systems. “Relevant risk factors” referred to tumor-related or patient-related factors that were components of these scoring systems or were repeatedly reported in the included studies in relation to renal ablation complications, including tumor diameter, proximity to the collecting system, central tumor location, history of myocardial infarction, and complicated diabetes mellitus. Studies were excluded if they met any of the following criteria: (1) they did not include patients undergoing renal ablation; (2) they did not report the R.E.N.A.L., PADUA, or (MC)^2^ score, or any prespecified related risk factor; (3) they did not report complications after renal ablation; (4) they provided insufficient data for extraction or quantitative synthesis; (5) they were reviews, systematic reviews, meta-analyses, editorials, letters, conference abstracts, meeting reports, expert opinions, case reports, or non-human studies; (6) they contained overlapping patient populations, in which case the most recent, largest, or most informative study was retained. “Insufficient data” was defined as the absence of extractable event numbers, odds ratios, confidence intervals, or other information sufficient to calculate an effect estimate for the association between a scoring system or risk factor and post-ablation complications.

The primary outcomes were the associations between the R.E.N.A.L., PADUA, and (MC)^2^ scoring categories and complications after renal ablation. Complications were analyzed as overall complications, major complications, and minor complications when data were available. Overall complications were defined as any adverse event occurring after renal ablation as reported by the original study. Major complications were defined as Clavien-Dindo grade ≥3 when the Clavien-Dindo classification was available. Minor complications were defined as Clavien-Dindo grade I–II or grade <3.

The secondary outcomes were the associations between individual risk factors or score components and post-ablation complications. These included maximum tumor diameter, exophytic/endophytic tumor properties, anterior/posterior tumor location, proximity to the collecting system, tumor location relative to the polar lines, central tumor location, history of myocardial infarction, and complicated diabetes mellitus.

Because the included studies differed in the duration and reporting of complication follow-up, we accepted the complication ascertainment period defined by each original study. When multiple time windows were reported, early post-procedural or 30-day complications were preferentially extracted for complication outcomes. The actual follow-up duration or complication ascertainment window for each study was extracted and summarized in the revised study characteristics table.

### 2.3 Data extraction

Two reviewers independently extracted data from all eligible studies using a predefined standardized data extraction form. The following information was extracted: first author, publication year, study period, study design, sample size, patient age, tumor stage when reported, tumor size, ablation modality, procedural approach, scoring system, complication outcomes, follow-up duration.

Disagreements between the two reviewers during data extraction were resolved by discussion and by rechecking the original full text and **Supplementary Materials**. If consensus could not be reached, a third reviewer adjudicated the disagreement. The final consensus data set was used for all meta-analyses.

### 2.4 Study quality assessment

The quality of the retrospective studies was assessed using the Newcastle-Ottawa Scale (NOS) (17). NOS scores range from 0 to 9, with scores greater than 6 considered indicative of high study quality.

### 2.5 Risk of bias assessment

The risk of bias in the included non-randomized studies was independently assessed by two reviewers using the ROBINS-I tool. For each study, the exposure was defined as the risk scoring category or risk factor under evaluation, and the outcome was post-ablation complications. The following seven domains were evaluated: bias due to confounding, bias in the selection of participants, bias in the classification of exposure/intervention, bias due to deviations from intended interventions, bias due to missing data, bias in outcome measurement, and bias in the selection of the reported results (18).

Each domain was judged as low, moderate, serious, or critical risk of bias, or as no information when judgment was not possible. The overall risk-of-bias judgment for each study was determined according to the highest level of risk identified in any domain. Disagreements were resolved through discussion and rechecking of the original article; unresolved disagreements were adjudicated by a third reviewer.

The ROBINS-I results were not used to determine statistical weights in the meta-analysis. Instead, they were used to evaluate the credibility of the evidence, identify potential methodological limitations, and guide the interpretation of pooled estimates. When sufficient studies were available, risk-of-bias judgments were considered in sensitivity analyses; otherwise, they were incorporated qualitatively into the Discussion and Limitations.

### 2.6 Data analysis

Statistical analyses were performed using Stata version 16.0 (StataCorp LLC, College Station, TX, USA) (19). Odds ratios (ORs) with 95% confidence intervals (CIs) were used as summary effect measures for dichotomous outcomes. A two-sided P value of less than 0.05 was considered statistically significant.

Statistical heterogeneity was assessed using Cochran’s chi-square test and the I^2^ statistic. I^2^ values of approximately 25%, 50%, and 75% were considered to indicate low, moderate, and high heterogeneity, respectively. A fixed-effect model was used when statistical heterogeneity was not significant, defined as I^2^ ≤50% and P ≥0.10 for Cochran’s chi-square test. A random-effects model was used when significant heterogeneity was present, defined as I^2^ >50% or P <0.10.

Because all included studies were observational and clinical heterogeneity was expected, statistical heterogeneity was interpreted together with clinical and methodological differences across studies. Potential sources of clinical heterogeneity included ablation modality, procedural approach, imaging guidance, tumor size, tumor location, tumor stage, scoring-system thresholds, patient comorbidities, complication definitions, complication classification systems, complication ascertainment windows, follow-up duration, and institutional or operator experience.

Subgroup analyses were planned, when sufficient data were available. Additional subgroup analyses according to ablation modality or complication severity were considered when data were sufficiently reported and comparable. Subgroup analysis was performed only when at least two studies were available in a subgroup. When the number of studies was insufficient or subgroup definitions were not comparable, formal subgroup analysis was not performed.

Sensitivity analyses were performed using a leave-one-out approach, in which one study was sequentially removed at a time to evaluate whether the pooled estimate was driven by any single study. Sensitivity analysis was performed for outcomes with a sufficient number of included studies. For outcomes based on only two studies, leave-one-out analysis was not considered meaningful and was therefore not performed.

Publication bias was assessed using funnel plots when at least 10 studies were available for an outcome. Egger’s test was also performed when the number of studies was sufficient. Publication-bias assessment was not performed for outcomes with fewer than 10 studies because funnel plots and statistical tests have limited reliability and low power in small meta-analyses.

## 3 Results

### 3.1 Description of study

**Figure 1:**
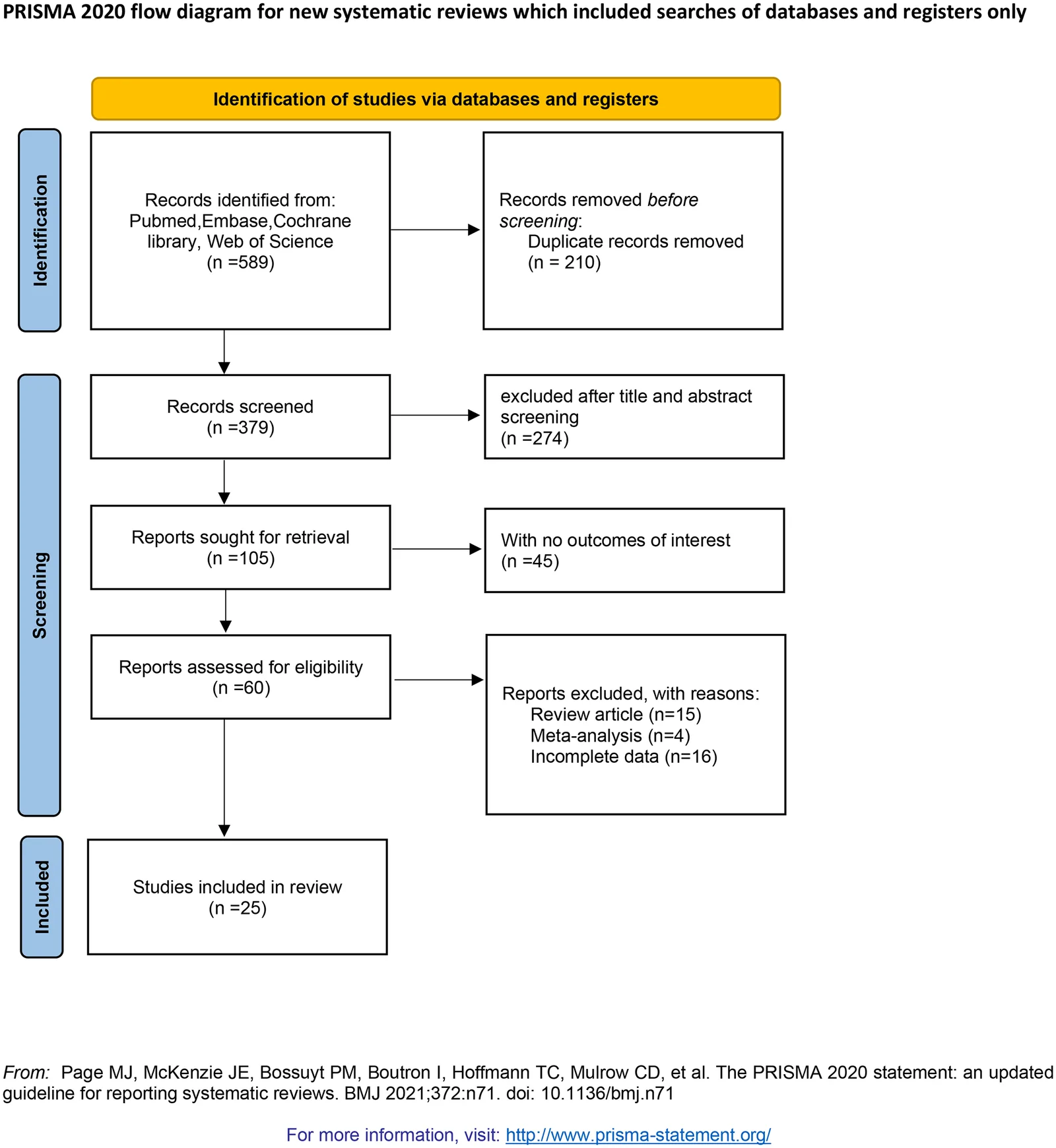
Flow diagram of the studies selection process.

A total of 589 records were identified from four databases. After 210 duplicate records were removed using reference management software, 274 studies were excluded after title and abstract screening. Of the remaining studies, 45 were excluded because they did not report outcomes of interest, 15 were systematic reviews, 4 were meta-analyses, and 16 had incomplete data. Ultimately, 25 studies involving 5,583 patients were included in this meta-analysis. The sample sizes ranged from 45 to 627 patients. All 25 included studies were retrospective (10–15, 20–38). The study selection process is shown in **Figure 1**, and the baseline characteristics of the included studies are presented in **Table 1**. The included studies were published between 2012 and 2025.

**Table 1:** Baseline data for studies included in the meta-analysis.

Auther	Year	Sample (n)	Type	Center	Study period	Age	Tumor size (cm)	Operation	Method
Sens, J.	2025	99	retrospective	single-center	2018-2023	70	NA	PCA	(MC)2
Evgenia Efthymiou	2023	76	retrospective	single-center	2013-2021	70.4	3	PMWA^a^	RENAL
Cody H. Savage	2023	116	retrospective	single-center	NA	67.8	2.3	PMWA	(MC)2
Gennaro Musi	2023	418	retrospective	single-center	2008-2021	67	2.6	PMWA/PRFA^b^	RENAL
Sienna Li	2022	116	retrospective	single-center	2004-2018	68.6	2.4	PMWA	RENAL
Benjamin J. McCafferty	2021	201	retrospective	single-center	2004-2019	65	2.8	PCA^c^	(MC)2
Jiapeng Wu	2020	211	retrospective	single-center	2006-2019	63.3	2.71	PMWA	RENAL
Seda Aladağ Kurt	2019	45	retrospective	single-center	NA	59.2	2.2	P/L RFA/CA	RENAL
Sepideh Shakeri	2019	56	retrospective	single-center	2013-2017	66	2.5	PMWA	RENAL
Kim A. Maciolek	2019	148	retrospective	single-center	2011-2017	67	2.4	PMWA	RENAL
Cristian Konstantinidis	2019	149	retrospective	single-center	2005-2015	71.7	2.6	PRFA	RENAL
Bimal Bhindi	2017	565	retrospective	single-center	2003-2015	71	3	PCA	RENAL
Anna Maria Ierardi	2017	58	retrospective	single-center	2008-2014	76.6	2.4	PMWA	RENAL
Marki E. Klapperich	2017	96	retrospective	single-center	2011-2015	66	2.6	PMWA	RENAL
O Rodriguez Faba	2016	80	retrospective	multicenter	2007-2013	66	2.6	PCA/LCA^d^	PADUA
Junlong Zhuang	2015	95	retrospective	single-center	2007-2013	53.1	2.6	LCA	PADUA
Xiaofeng Chang	2015	215	retrospective	Multi-center	2006-2014	55.6	3.6	LRFA^e^	RENAL
Brunolf W Lagerveld	2014	97	retrospective	single-center	2006-2013	66.4	3	LCA	RENAL PADUA
Grant D Schmit	2014	375	retrospective	single-center	2003-2011	69.7	3.2	PCA	(MC)2
David M Sisul	2013	154	retrospective	Multi-center	2003-2011	68	2.6	LCA/PCA	RENAL PADUA
Jose Reyes	2013	39	retrospective	single-center	NA	71	2	LCA/PCA/PRFA	RENAL
Casey A Seideman	2013	199	retrospective	single-center	2001-2011	67	2.4	LRFA/PRFA	RENAL
Michael L Blute Jr	2013	139	retrospective	single-center	2005-2011	70	2.4	PCA	RENAL PADUA
Grant D Schmit	2013	627	retrospective	single-center	2000-2012	68.7	2.7	PCA/PRFA	RENAL
Zhamshid Okhunov	2012	210	retrospective	Multi-center	2005-2010	64.5	2.6	LCA	RENAL

^a^ PMWA, Percutaneous microwave ablation.

^b^ PRFA, Percut.

### 3.2 Quality assessment

The quality of the included cohort studies was evaluated using the Newcastle-Ottawa Scale (NOS), with scores ranging from 6 to 8. All 25 studies included in the review had NOS scores of 6 or higher, four of which were of moderate quality, 21 studies were of high quality (**Table 2**).

**Table 2:** Quality score of included studies based on the NOS scale.

Author(year)	Selection			Comparability			Exposure			Total stars
	^a^REC	^b^SNEC	^c^AE	^d^DO	^e^SC	^f^AF	^g^AO	^h^FU	^i^AFU	
Sens, J (15)	1	1	1	1	1		1		1	7
Evgenia Efthymiou (20)	1	1	1	1	1	1	1			7
Cody H. Savage (21)	1	1	1	1		1		1		6
Gennaro Musi (22)	1	1	1	1	1		1	1		7
Sienna Li (14)	1	1	1	1			1	1	1	7
Benjamin J. McCafferty (23)	1	1	1		1	1	1	1		7
Jiapeng Wu (24)	1	1	1	1	1		1		1	7
Seda Aladağ Kurt (25)	1	1	1	1				1	1	6
Sepideh Shakeri (26)	1	1	1	1	1	1	1			7
Kim A. Maciolek (27)	1	1	1	1		1	1	1		7
Cristian Konstantinidis (28)	1	1	1		1		1		1	6
Bimal Bhindi (13)	1	1	1			1	1	1	1	7
Anna Maria Ierardi (29)	1	1	1	1			1	1	1	7
Marki E. Klapperich (30)	1	1	1	1	1	1	1	1		8
O Rodriguez Faba (31)	1	1	1	1	1	1	1			7
Junlong Zhuang (32)	1	1	1	1	1				1	6
Xiaofeng Chang (11)	1	1	1	1			1	1	1	7
Brunolf W Lagerveld (33)	1	1	1	1		1	1	1		7
Grant D Schmit (10)	1	1	1	1	1	1	1	1		8
David M Sisul (34)	1	1	1	1	1	1	1			7
Jose Reyes (35)	1	1	1	1		1	1	1		7
Casey A Seideman (36)	1	1	1	1	1	1	1			7
Michael L Blute Jr (37)	1	1	1	1			1	1	1	7
Grant D Schmit (10)	1	1	1	1	1			1	1	7
Zhamshid Okhunov (38)	1	1	1	1		1	1	1	1	8

^a^ REC, representativeness of the cohort.

^b^ SNEC, selection of the none posed cohort.

^c^ AE, ascertainment of exposure.

^d^ DO, demonstration that outcome of interest was not present at start of study.

^e^ SC, study controls most important factors.

^f^ AF, study controls for other important factors.

^g^ AO, assessment of outcome.

^h^ FU, follow-up long enough for outcomes to occur.

^i^ AFU, adequacy of follow-up of cohort (≥ 80%).

Risk of bias was further evaluated using ROBINS-I. Overall, 25 studies were judged to have moderate risk of bias. No study was judged to have critical risk of bias. Detailed ROBINS-I judgments are presented in **Supplementary Table 2**.

### 3.3 R.E.N.A.L. scoring system

Fifteen studies reported the association between R.E.N.A.L. score categories and overall complications. In the comparison between the low-risk group and the intermediate-risk group, the intermediate-risk group was used as the reference group. The low-risk group had significantly lower odds of overall complications than the intermediate-risk group (OR = 0.45, 95% CI 0.27–0.75, P<0.05; I^2^=48.4%, P_heterogeneity=0.018; random-effects model). Thus, the result was consistent with higher odds of complications in the intermediate-risk group.

**Figure 2:**
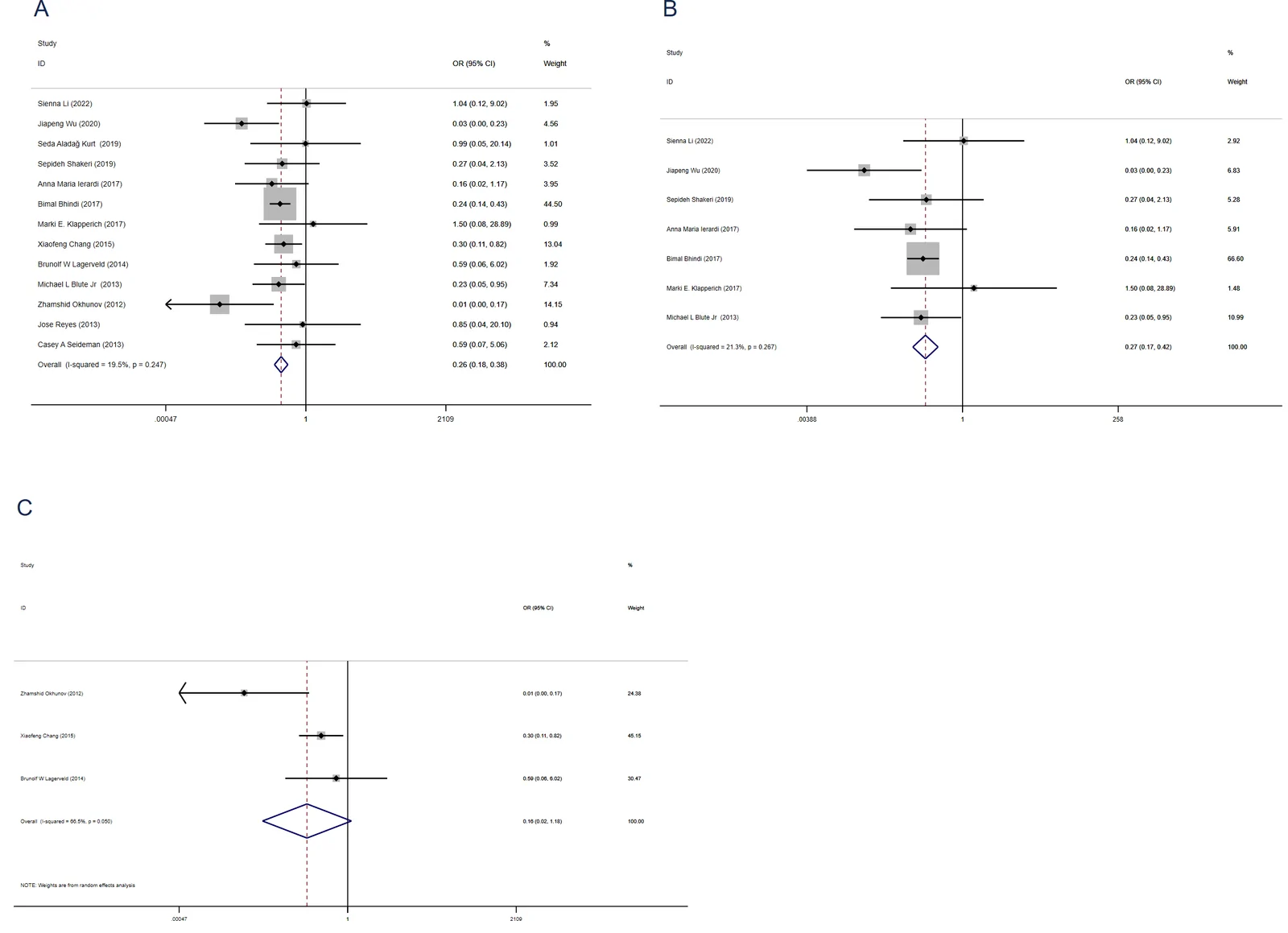
Forest plots of the association between low-risk and intermediate-risk R.E.N.A.L. score categories and overall complications after renal ablation. **(A)** Overall analysis comparing the low-risk group with the intermediate-risk group. **(B)** Percutaneous ablation subgroup. **(C)** Laparoscopic ablation subgroup. The intermediate-risk group was used as the reference category. OR<1 indicates lower odds of overall complications in the low-risk group relative to the intermediate-risk group. The solid vertical line represents the null effect value of OR = 1, and the red dashed vertical line represents the pooled effect estimate. Truncated confidence intervals are indicated by arrows.

In the percutaneous ablation subgroup, using the intermediate-risk group as the reference, the pooled estimate did not reach statistical significance (8 studies; OR = 0.51, 95% CI 0.23–1.13, P = 0.10; I^2^=55.0%, P_heterogeneity=0.029; random-effects model). Similarly, the laparoscopic subgroup estimate was not statistically significant (3 studies; OR = 0.29, 95% CI 0.05–1.75, P = 0.18; I^2^=78.8%, P_heterogeneity=0.009; random-effects model). Given the small number of studies, wide confidence intervals, and substantial heterogeneity, subgroup findings should be interpreted cautiously (**Figure 2**).

Thirteen studies compared the intermediate-risk group with the high-risk group, with the high-risk group used as the reference. The intermediate-risk group had significantly lower odds of overall complications than the high-risk group (OR = 0.26, 95% CI 0.18–0.38, P<0.05; I^2^=19.5%, P_heterogeneity=0.247; fixed-effect model). A similar association was observed in the percutaneous ablation subgroup (7 studies; OR = 0.27, 95% CI 0.17–0.42, P<0.05; I^2^=21.3%, P_heterogeneity=0.267; fixed-effect model).

**Figure 3:**
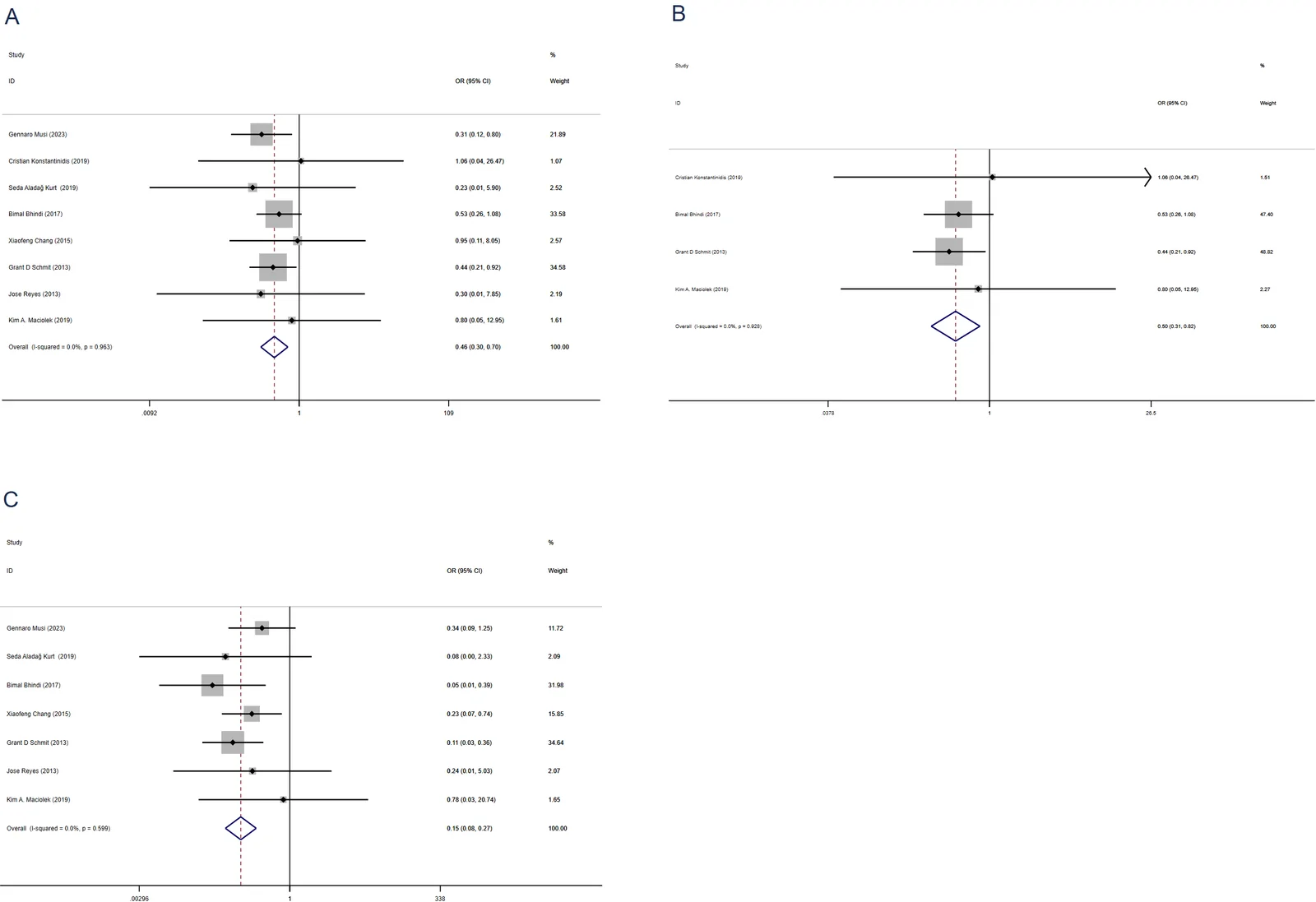
Forest plots of the association between intermediate-risk and high-risk R.E.N.A.L. score categories and overall complications after renal ablation. **(A)** Overall analysis comparing the intermediate-risk group with the high-risk group. **(B)** Percutaneous ablation subgroup. **(C)** Laparoscopic ablation subgroup. The high-risk group was used as the reference category. OR<1 indicates lower odds of overall complications in the intermediate-risk group relative to the high-risk group. The solid vertical line represents the null effect value of OR = 1, and the red dashed vertical line represents the pooled effect estimate. Truncated confidence intervals are indicated by arrows.

In contrast, the laparoscopic subgroup estimate did not reach statistical significance (3 studies; OR = 0.16, 95% CI 0.02–1.18, P = 0.07; I^2^=66.5%, P_heterogeneity=0.050; random-effects model). This non-significant subgroup result should not be interpreted as definitive evidence of no association because it was based on only three heterogeneous studies (**Figure 3**).

**Figure 4:**
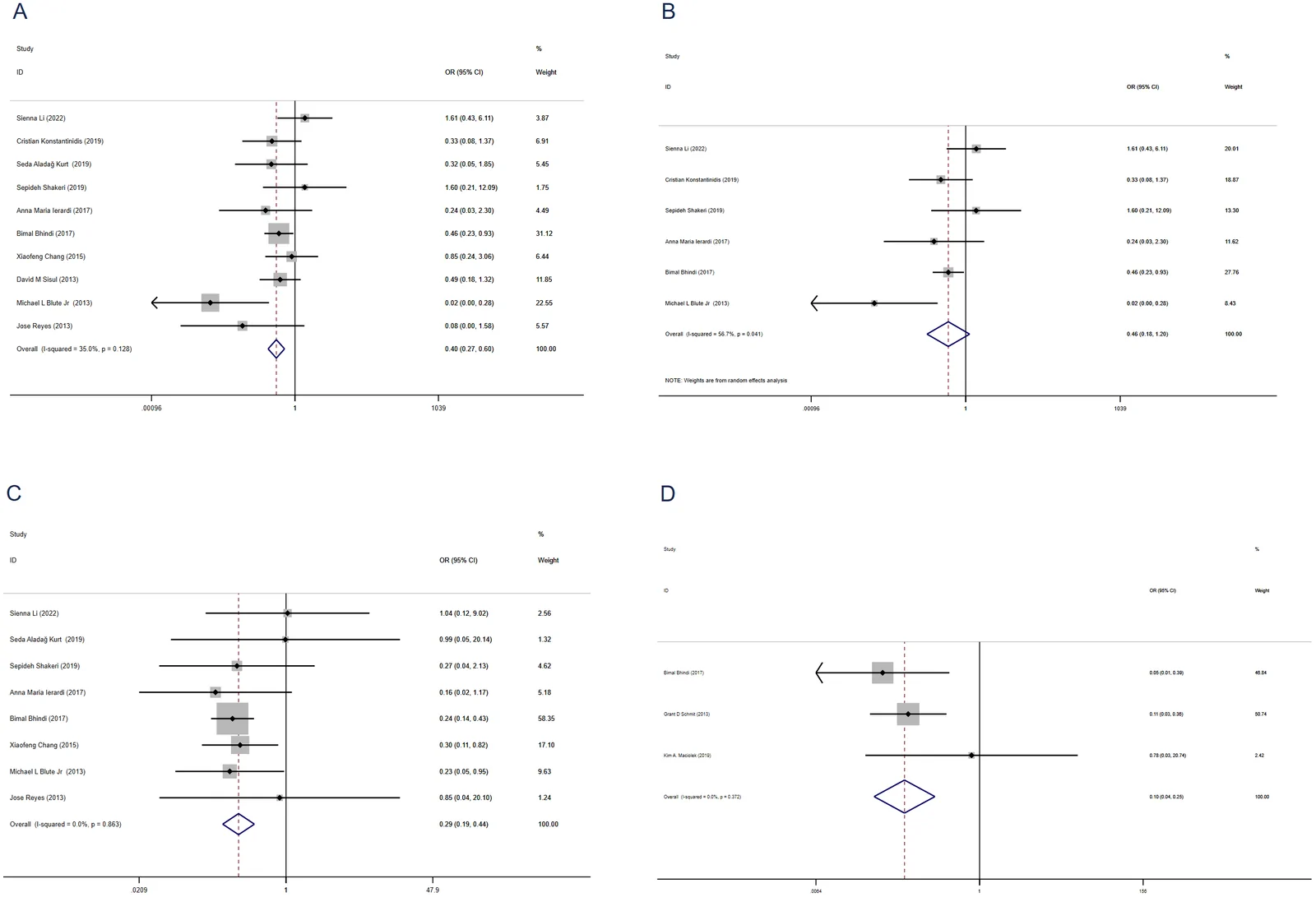
Forest plots of the association between R.E.N.A.L. score categories and major complications after renal ablation. Major complications were defined as Clavien-Dindo grade ≥3 when this classification was available. **(A)** Overall analysis comparing the low-risk group with the intermediate-risk group. **(B)** Percutaneous ablation subgroup for the low-risk versus intermediate-risk comparison. **(C)** Overall analysis comparing the intermediate-risk group with the high-risk group. **(D)** Percutaneous ablation subgroup for the intermediate-risk versus high-risk comparison. In **(A, B)**, the intermediate-risk group was used as the reference category. In **(C, D)**, the high-risk group was used as the reference category. OR<1 indicates lower odds of major complications in the first-listed group relative to the reference group. The solid vertical line represents the null effect value of OR = 1, and the red dashed vertical line represents the pooled effect estimate. Truncated confidence intervals are indicated by arrows.

Eight studies reported major complications, defined as Clavien–Dindo grade ≥3 when this classification was available. Using the intermediate-risk group as the reference, the low-risk group had significantly lower odds of major complications (OR = 0.46, 95% CI 0.30–0.70, P<0.05; I^2^=0.0%, P_heterogeneity=0.963; fixed-effect model). The corresponding percutaneous ablation subgroup analysis showed a similar result (4 studies; OR = 0.50, 95% CI 0.31–0.82, P<0.05; I^2^=0.0%, P_heterogeneity=0.928; fixed-effect model). Seven studies compared the intermediate-risk group with the high-risk group, with the high-risk group serving as the reference. The intermediate-risk group had significantly lower odds of major complications (OR = 0.15, 95% CI 0.08–0.27, P<0.05; I^2^=0.0%, P_heterogeneity=0.599; fixed-effect model). This association was also observed in the percutaneous ablation subgroup (3 studies; OR = 0.10, 95% CI 0.04–0.25, P<0.05; I^2^=0.0%, P_heterogeneity=0.372; fixed-effect model) (**Figure 4**). A pooled laparoscopic estimate was not presented because the available laparoscopic data were insufficient for quantitative subgroup synthesis (**Figure 4**).

Ten studies reported minor complications, defined as Clavien–Dindo grade <3 when this classification was available. Using the intermediate-risk group as the reference, the low-risk group had significantly lower odds of minor complications (OR = 0.40, 95% CI 0.27–0.60, P<0.05; I^2^=35.0%, P_heterogeneity=0.128; fixed-effect model).

The percutaneous subgroup estimate did not reach statistical significance (6 studies; OR = 0.46, 95% CI 0.18–1.20, P = 0.11; I^2^=56.7%, P_heterogeneity=0.041; random-effects model). This finding should be interpreted cautiously because of the limited number of studies and moderate heterogeneity.

**Figure 5:**
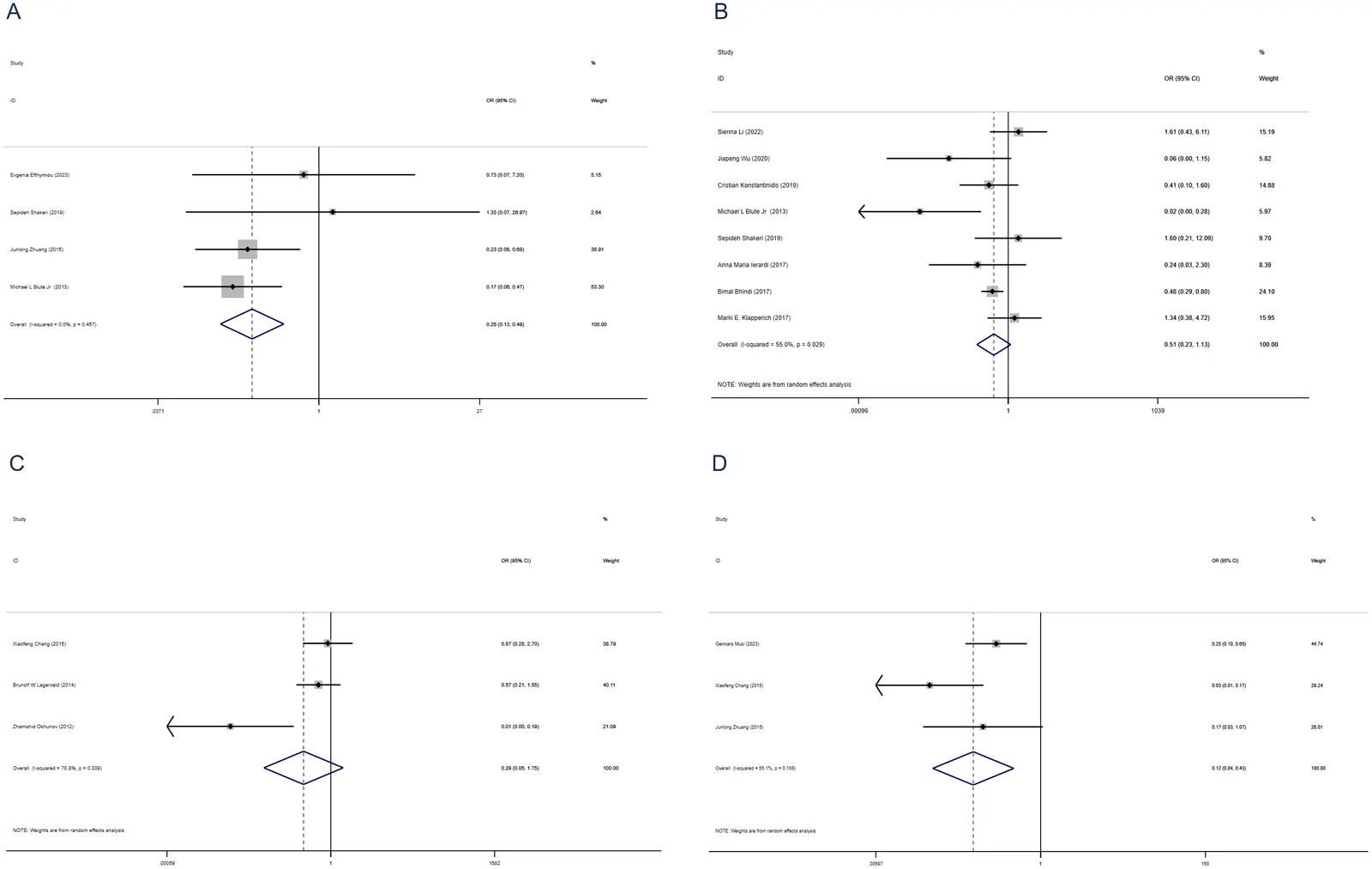
Forest plots of the association between R.E.N.A.L. score categories and minor complications after renal ablation. Minor complications were defined as Clavien-Dindo grade <3 when this classification was available. **(A)** Overall analysis comparing the low-risk group with the intermediate-risk group. **(B)** Percutaneous ablation subgroup for the low-risk versus intermediate-risk comparison. **(C)** Overall analysis comparing the intermediate-risk group with the high-risk group. **(D)** Percutaneous ablation subgroup for the intermediate-risk versus high-risk comparison. In **(A, B)**, the intermediate-risk group was used as the reference category. In **(C, D)**, the high-risk group was used as the reference category. OR<1 indicates lower odds of minor complications in the first-listed group relative to the reference group. The solid vertical line represents the null effect value of OR = 1, and the red dashed vertical line represents the pooled effect estimate. Truncated confidence intervals are indicated by arrows.

Eight studies compared the intermediate-risk group with the high-risk group, with the high-risk group used as the reference. The intermediate-risk group had significantly lower odds of minor complications (OR = 0.29, 95% CI 0.19–0.44, P<0.05; I^2^=0.0%, P_heterogeneity=0.863; fixed-effect model). The association remained statistically significant in the percutaneous ablation subgroup (5 studies; OR = 0.26, 95% CI 0.16–0.42, P<0.05; I^2^=0.0%, P_heterogeneity=0.749; fixed-effect model) (**Figure 5**). Laparoscopic subgroup pooling was not performed because insufficient studies provided extractable data for these comparisons (**Figure 5**).

**Figure 6:**
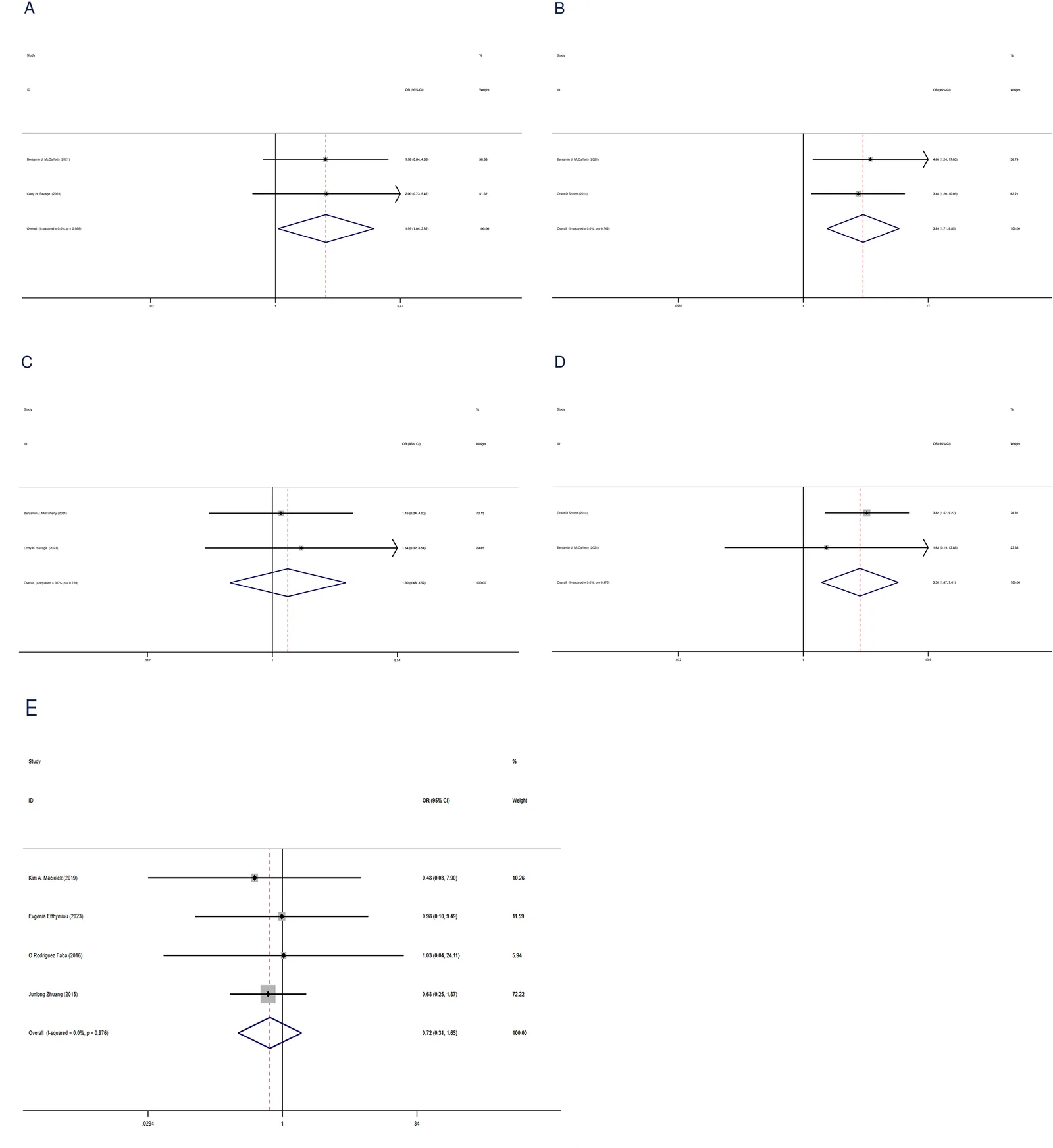
Forest plots of the association between individual R.E.N.A.L. score components and overall complications after renal ablation. **(A)** Maximum tumor diameter, comparing tumors measuring ≤4 cm with tumors measuring >4 cm; tumors >4 cm were used as the reference category. **(B)** Exophytic/endophytic tumor component, comparing [insert comparison category] with [insert reference category]. **(C)** Anterior/posterior tumor location, comparing [insert comparison category] with [insert reference category]. **(D)** Proximity to the collecting system, comparing tumors closer to the collecting system with tumors farther from the collecting system; tumors farther from the collecting system were used as the reference category. **(E)** Tumor location relative to the polar lines, comparing [insert comparison category] with [insert reference category]. OR<1 indicates lower odds of complications in the comparison group relative to the reference group, whereas OR>1 indicates higher odds of complications in the comparison group. The solid vertical line represents the null effect value of OR = 1, and the red dashed vertical line represents the pooled effect estimate. Truncated confidence intervals are indicated by arrows.

Four studies evaluated maximum tumor diameter. Using tumors measuring >4 cm as the reference group, tumors measuring ≤4 cm had significantly lower odds of complications (OR = 0.25, 95% CI 0.13–0.49, P<0.05; I^2^=0.0%, P_heterogeneity=0.457; fixed-effect model). This finding was consistent with higher complication odds among tumors measuring >4 cm. Three studies evaluated the exophytic/endophytic component of the R.E.N.A.L. score. The pooled estimate showed no statistically significant association between the exophytic/endophytic component and complications (OR = 1.41, 95% CI 0.35–5.67, P = 0.63; I^2^=0.0%, P_heterogeneity=0.753; fixed-effect model). hree studies evaluated anterior/posterior tumor location. The pooled estimate showed no statistically significant association between anterior/posterior location and complications (OR = 0.25, 95% CI 0.04–1.69, P = 0.16; I^2^=75.1%, P_heterogeneity=0.018; random-effects model). hree studies evaluated tumor proximity to the collecting system. Using tumors located farther from the collecting system as the reference group, tumors located closer to the collecting system had significantly higher odds of complications (OR = 3.15, 95% CI 1.39–7.12, P<0.05; I^2^=13.3%, P_heterogeneity=0.316; fixed-effect model). Four studies evaluated tumor location relative to the polar lines. The pooled estimate showed no statistically significant association between tumor location relative to the polar lines and complications (OR = 0.72, 95% CI 0.31–1.65, P = 0.44; I^2^=0.0%, P_heterogeneity=0.976; fixed-effect model). Because each component-level analysis included only three or four studies, these results should be interpreted as exploratory. (**Figure 6**).

### 3.4 PADUA scoring system

**Figure 7:**
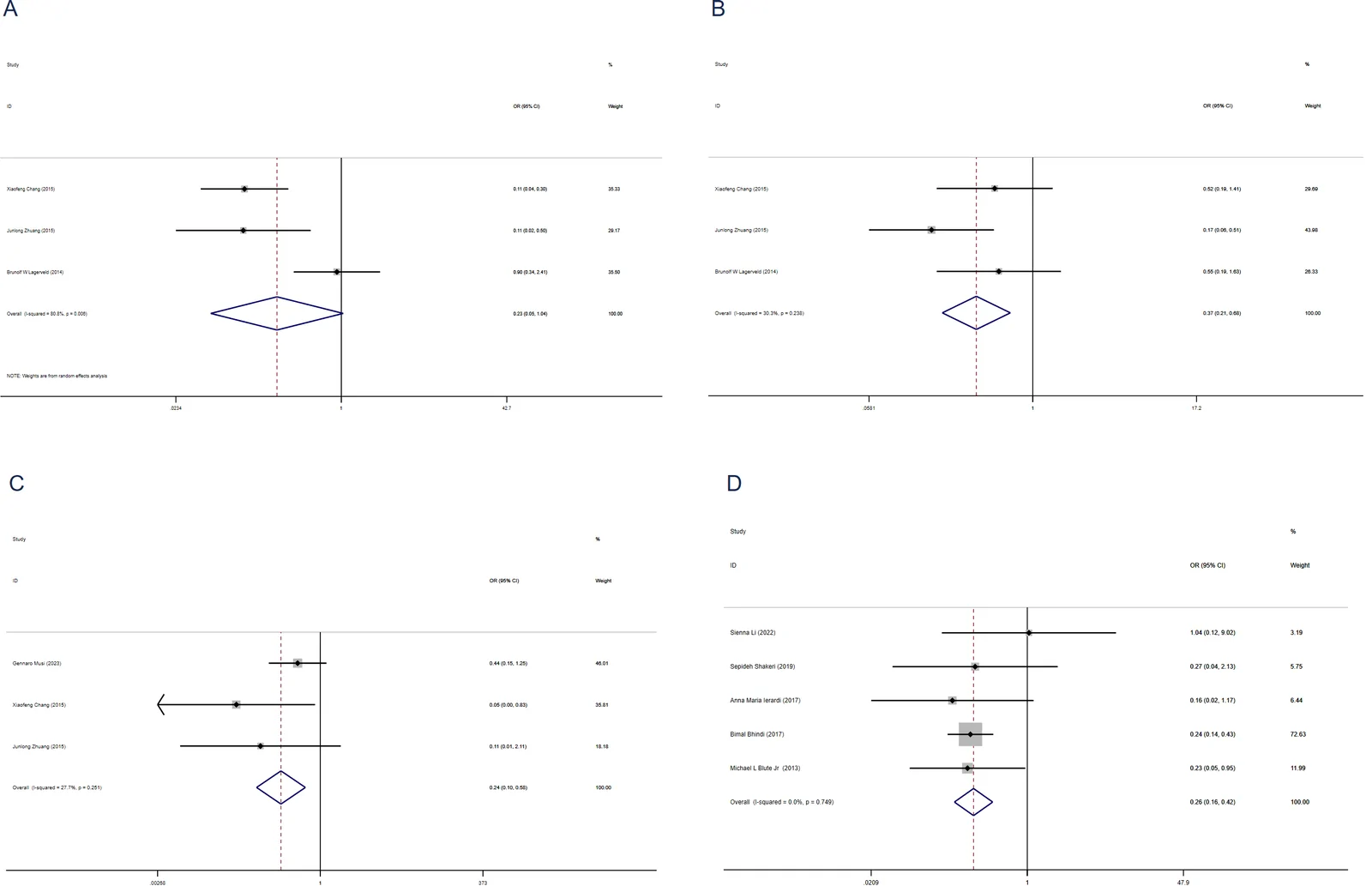
Forest plots of the association between PADUA score categories and complications after renal ablation. **(A)** Overall complications, comparing the low-risk group with the intermediate-risk group. **(B)** Overall complications, comparing the intermediate-risk group with the high-risk group. **(C)** Major complications, comparing the low-risk group with the intermediate-risk group. **(D)** Major complications, comparing the intermediate-risk group with the high-risk group. Major complications were defined as Clavien-Dindo grade ≥3 when this classification was available. In **(A, C)**, the intermediate-risk group was used as the reference category. In **(B, D)**, the high-risk group was used as the reference category. OR<1 indicates lower odds of complications in the first-listed group relative to the reference group. The solid vertical line represents the null effect value of OR = 1, and the red dashed vertical line represents the pooled effect estimate. Truncated confidence intervals are indicated by arrows.

Three studies reported associations between PADUA score categories and overall complications. For the comparison between the low-risk group (PADUA score <8) and the intermediate-risk group (PADUA score 8–9), the intermediate-risk group was used as the reference. The pooled estimate did not reach statistical significance (OR = 0.23, 95% CI 0.05–1.04, P = 0.06; I^2^=80.8%, P_heterogeneity=0.006; random-effects model). For the comparison between the intermediate-risk and high-risk groups, the high-risk group was used as the reference. The intermediate-risk group had significantly lower odds of overall complications than the high-risk group (OR = 0.37, 95% CI 0.21–0.68, P<0.05; I^2^=30.3%, P_heterogeneity=0.238; fixed-effect model). Three studies reported major complications. Using the intermediate-risk group as the reference, the low-risk group had significantly lower odds of major complications (OR = 0.24, 95% CI 0.10–0.58, P<0.05; I^2^=27.7%, P_heterogeneity=0.251; fixed-effect model). Using the high-risk group as the reference, the intermediate-risk group also had significantly lower odds of major complications (OR = 0.12, 95% CI 0.04–0.43, P<0.05; I^2^=55.1%, P_heterogeneity=0.108; random-effects model). Because all PADUA analyses included only three studies, these findings should be interpreted cautiously (**Figure 7**).

### 3.5 (MC)2 risk scoring system

**Figure 8:**
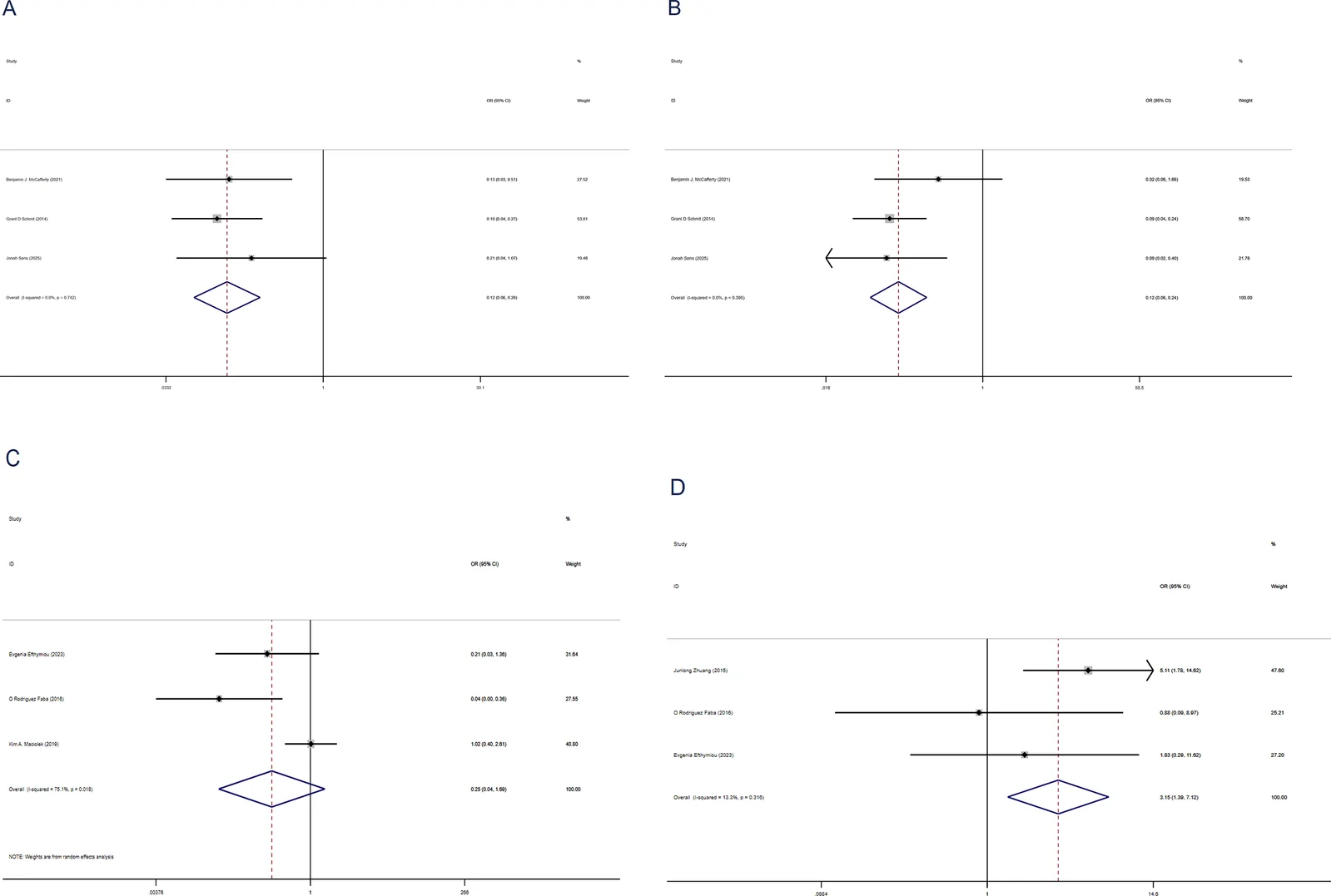
Forest plots of the association between (MC)^2^ risk score categories and major complications after renal ablation. Major complications were defined as Clavien-Dindo grade ≥3 when this classification was available. **(A)** Comparison between the low-risk group and the intermediate-risk group. The intermediate-risk group was used as the reference category. **(B)** Comparison between the intermediate-risk group and the high-risk group. The high-risk group was used as the reference category. OR<1 indicates lower odds of major complications in the first-listed group relative to the reference group. The solid vertical line represents the null effect value of OR = 1, and the red dashed vertical line represents the pooled effect estimate. Truncated confidence intervals are indicated by arrows.

Four studies evaluated the association between the (MC)^2^ risk score and major complications. In the comparison between the low-risk group [(MC)^2^ score <5] and the intermediate-risk group [(MC)^2^ score 5–8], the intermediate-risk group was used as the reference. The low-risk group had significantly lower odds of major complications (3 studies; OR = 0.12, 95% CI 0.06–0.26, P<0.05; I^2^=0.0%, P_heterogeneity=0.742; fixed-effect model). In the comparison between the intermediate-risk group and the high-risk group [(MC)^2^ score >8], the high-risk group was used as the reference. The intermediate-risk group had significantly lower odds of major complications (3 studies; OR = 0.12, 95% CI 0.06–0.24, P<0.05; I^2^=0.0%, P_heterogeneity=0.395; fixed-effect model). Because these analyses were based on only three studies, the findings should be interpreted cautiously (**Figure 8**).

**Figure 9:**
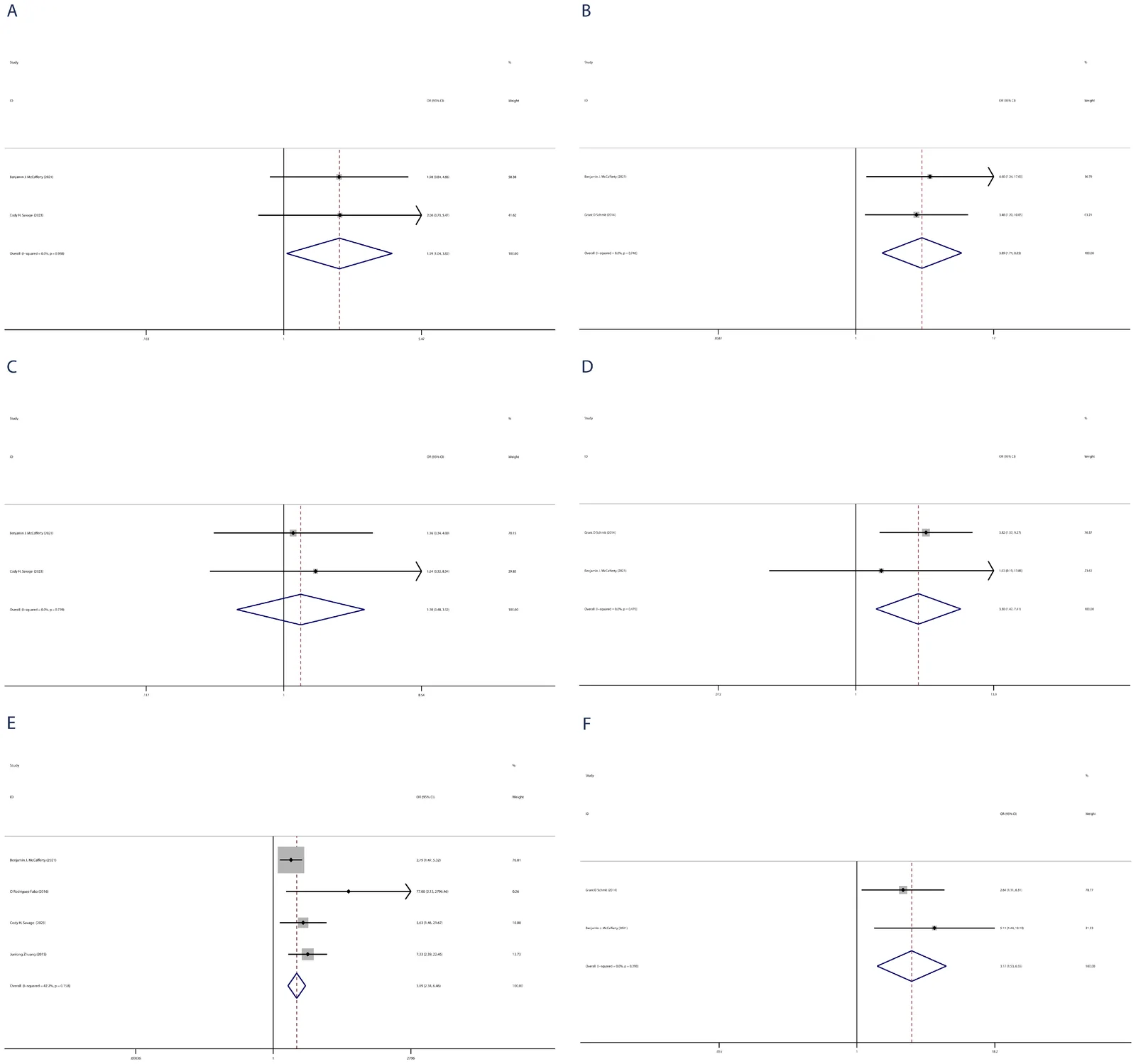
Forest plots of the association between selected (MC)^2^-related risk factors and complications after renal ablation. **(A)** Complicated diabetes mellitus and overall complications. **(B)** Complicated diabetes mellitus and major complications. **(C)** History of myocardial infarction and overall complications. **(D)** History of myocardial infarction and major complications. **(E)** Central tumor location and overall complications. **(F)** Central tumor location and major complications. For panels **(A–D)**, patients without the corresponding risk factor were used as the reference category. For panels E and F, non-central tumors were used as the reference category. OR>1 indicates higher odds of complications in patients with the risk factor or in tumors with central location. The solid vertical line represents the null effect value of OR = 1, and the red dashed vertical line represents the pooled effect estimate. Truncated confidence intervals are indicated by arrows. DM, diabetes mellitus; MI, myocardial infarction.

Three studies reported complicated diabetes mellitus. Compared with patients without complicated diabetes mellitus, patients with complicated diabetes mellitus had higher odds of overall complications in an exploratory pooled analysis (2 studies; OR = 1.99, 95% CI 1.04–3.82, P<0.05; I^2^=0.0%, P_heterogeneity=0.968; fixed-effect model). Complicated diabetes mellitus was also associated with higher odds of major complications (2 studies; OR = 3.89, 95% CI 1.71–8.85, P<0.05; I^2^=0.0%, P_heterogeneity=0.746; fixed-effect model). Three studies reported a history of myocardial infarction. Compared with patients without a history of myocardial infarction, those with a history of myocardial infarction did not have significantly different odds of overall complications (2 studies; OR = 1.30, 95% CI 0.48–3.52, P = 0.60; I^2^=0.0%, P_heterogeneity=0.739; fixed-effect model). However, a history of myocardial infarction was associated with higher odds of major complications (2 studies; OR = 3.30, 95% CI 1.47–7.41, P<0.05; I^2^=0.0%, P_heterogeneity=0.470; fixed-effect model). Four studies evaluated central tumor location. Compared with non-central tumors, centrally located tumors were associated with higher odds of overall complications (4 studies; OR = 3.89, 95% CI 2.34–6.46, P<0.05; I^2^=42.2%, P_heterogeneity=0.158; fixed-effect model). Central tumor location was also associated with higher odds of major complications (2 studies; OR = 3.17, 95% CI 1.53–6.55, P<0.05; I^2^=0.0%, P_heterogeneity=0.398; fixed-effect model). Because several analyses of complicated diabetes mellitus, myocardial infarction, and central tumor location included only two or three studies, these results should be regarded as exploratory and hypothesis-generating rather than definitive evidence of independent risk factors (**Figure 9**).

## 4 Sensitivity analysis

Sensitivity analyses were performed to explore potential sources of heterogeneity for each outcome. The results suggested that the pooled estimates for the PADUA scoring system and central tumor location were relatively stable, indicating that these findings were not substantially influenced by any single study.

## 5 Publication bias

Publication bias was assessed for the R.E.N.A.L. scoring system outcomes. Visual inspection of the funnel plots suggested some asymmetry. Although the included studies showed a generally dispersed distribution, the possibility of publication bias could not be excluded (**Supplementary Figure S1**).

## 6 Discussion

This meta-analysis synthesized the available evidence regarding the associations of the R.E.N.A.L., PADUA, and (MC)^2^ scoring systems with complications after renal ablation. The principal findings were that higher categories of all three scoring systems were generally associated with greater odds of major complications. Higher R.E.N.A.L. score categories were also associated with overall and minor complications in several pooled comparisons. Among individual anatomical components, larger tumor diameter and closer proximity to the collecting system were associated with complications, whereas exophytic/endophytic properties, anterior/posterior location, and location relative to the polar lines were not significantly associated with complications. Exploratory analyses also suggested possible associations of central tumor location, complicated diabetes mellitus, and a history of myocardial infarction with selected complication outcomes. However, all included studies were retrospective, and the PADUA, (MC)^2^, and several individual risk-factor analyses were based on only two to four studies. The findings should therefore be interpreted as associations rather than evidence of causation or definitive predictive performance.

The R.E.N.A.L. score was evaluated in the largest number of included studies. The pooled results suggested a gradient of increasing complication risk across adjacent R.E.N.A.L. categories, particularly for major complications. Nevertheless, the findings were not entirely consistent across procedural subgroups. For example, although some overall analyses were statistically significant, the corresponding percutaneous or laparoscopic subgroup estimates did not always reach statistical significance. These subgroup findings should not be interpreted as evidence that no association exists within a particular procedural approach. Stratification reduced the number of contributing studies and statistical power, and several subgroup estimates had wide confidence intervals or substantial heterogeneity. Moreover, a statistically significant result in one subgroup and a non-significant result in another does not establish a difference between subgroups in the absence of a formal interaction test.

The association between higher R.E.N.A.L. scores and complications is clinically plausible because higher scores generally reflect greater anatomical complexity. Tumors in higher-risk categories tend to be larger, more central, or closer to the collecting system and renal sinus, potentially increasing the technical difficulty of probe placement and complete tumor coverage (14, 26). Such tumors may require additional applicators, overlapping ablation zones, wider treatment margins, or longer energy delivery, thereby increasing the possibility of injury to the collecting system, renal parenchyma, or adjacent vessels (11, 13, 37). However, this biological plausibility should not be regarded as proof that the score independently predicts complications, because adjustment for patient characteristics, tumor characteristics, ablation technique, and operator-related factors was inconsistent across the included studies. Minor complications may also be particularly sensitive to institutional reporting thresholds and perioperative management, which may partly explain why their association with anatomical complexity was less consistent than that observed for major complications (11–13, 22).

The PADUA score showed a broadly similar direction of association, particularly for major complications. However, all PADUA analyses included only three studies. The low-risk versus intermediate-risk comparison for overall complications did not reach statistical significance and showed substantial heterogeneity, whereas the intermediate-risk versus high-risk comparison was statistically significant. Both adjacent-category comparisons were significant for major complications. These findings suggest that greater PADUA-defined anatomical complexity may be associated with clinically important complications, but they do not constitute a robust validation of the PADUA score for renal ablation. High PADUA categories reflect features such as larger tumor size, deeper location, and proximity to the renal sinus or collecting system, which may increase the likelihood of vascular or urinary tract injury during ablation (39, 40). Nevertheless, the small evidence base, wide confidence intervals, and differences among the included studies substantially limit the precision and generalizability of these estimates.

The analyses of individual anatomical characteristics provide additional context for the scoring-system findings. Larger tumors and tumors closer to the collecting system were associated with greater odds of complications. Larger tumors may require more extensive or overlapping treatment zones, whereas tumors close to the collecting system may increase the risk of thermal or cryogenic injury to the calyces, renal pelvis, or proximal ureter (41). By contrast, exophytic/endophytic properties, anterior/posterior location, and location relative to the polar lines were not significantly associated with complications. These non-significant findings should not be interpreted as evidence that these characteristics have no clinical relevance, because each analysis included only three or four studies, some confidence intervals were wide, and the anterior/posterior analysis showed substantial heterogeneity.

The (MC)^2^ score differs conceptually from R.E.N.A.L. and PADUA because it was developed specifically for renal ablation and incorporates both tumor-related and patient-related factors, including maximum tumor diameter, central tumor location, a history of myocardial infarction, and complicated diabetes mellitus10. Higher (MC)^2^ categories were associated with greater odds of major complications in the pooled analyses. However, each comparison included only three studies, and the similar pooled point estimates for the two adjacent-category comparisons reflected sparse data. Therefore, the current findings suggest a potential association but do not establish the discriminative performance or incremental clinical value of the (MC)^2^ score.

The analyses of complicated diabetes mellitus, myocardial infarction, and central tumor location should be interpreted with particular caution. Associations of complicated diabetes mellitus with overall and major complications and of myocardial infarction with major complications were each based on only two studies. Similarly, the analysis of central tumor location and major complications included only two studies. These findings are clinically plausible: central tumors are more likely to be adjacent to the renal sinus, hilar vessels, and collecting system7; complicated diabetes mellitus may be associated with impaired tissue healing, microvascular disease, infection susceptibility, and a greater comorbidity burden (42, 43); and a history of myocardial infarction may reflect limited cardiovascular reserve or exposure to antiplatelet or anticoagulant treatment. Nevertheless, the available analyses do not demonstrate that these variables are independent risk factors. They should be regarded as hypothesis-generating observations requiring validation in adequately adjusted prospective cohorts.

The three scoring systems should not be interpreted as forming a hierarchy of superiority. The R.E.N.A.L. score was evaluated in more studies, PADUA provided another anatomy-based assessment, and the (MC)^2^ score incorporated both anatomical and comorbidity-related variables. These differences describe the design and current evidence base of the scoring systems rather than comparative performance. No direct head-to-head meta-analysis was performed, and the available studies did not consistently report comparative measures of discrimination, calibration, or clinical net benefit. Consequently, the present analysis cannot determine whether one scoring system performs better than another or whether combining anatomical and patient-related factors provides incremental predictive value.

In clinical practice, these scores may provide structured information for preprocedural risk assessment, patient counselling, procedural planning, and postoperative monitoring. Higher-risk categories may alert clinicians to cases requiring more detailed imaging review, adjunctive protective measures, multidisciplinary discussion, or closer observation after ablation. However, these scores should be regarded as supportive risk-stratification tools rather than stand-alone criteria for selecting ablation, surgery, or active surveillance. Their application should remain integrated with patient age, comorbidity, renal function, tumor characteristics, treatment goals, operator experience, and institutional expertise.

Several sources of clinical and methodological heterogeneity should be considered. The included studies differed in ablation modality, procedural approach, imaging guidance, tumor stage, patient selection, tumor complexity, operator experience, and institutional practice. Radiofrequency ablation, microwave ablation, and cryoablation differ in energy delivery and ablation-zone formation, whereas percutaneous and laparoscopic procedures may involve different patterns of tumor selection, accessibility, anesthesia, and perioperative monitoring. Complication definitions and classification systems also varied, and follow-up duration and complication ascertainment windows were not standardized. Exact event-level timing of complications was not consistently available. These differences may affect both the observed complication rate and the likelihood of detecting minor or delayed events. The use of random-effects models accounts statistically for some between-study variability but does not eliminate clinical heterogeneity or bias.

The quality and risk-of-bias assessments further limit the strength of the conclusions. Although NOS scores indicated moderate-to-high methodological quality, a relatively high NOS score does not exclude important bias. ROBINS-I identified concerns related to residual confounding, retrospective participant selection, incomplete adjustment for baseline patient and tumor characteristics, and variation in complication measurement or reporting. Because all included studies were retrospective observational studies, the pooled estimates represent associations and should not be interpreted as causal effects.

This study has several additional limitations. First, the evidence base was sparse for PADUA, (MC)^2^, and several individual risk factors, and some pooled estimates were derived from only two studies. Such analyses may be unstable and were not suitable for meaningful leave-one-out sensitivity analysis or publication-bias assessment. Second, the aggregate data did not permit consistent adjustment for potentially important confounders, including age, comorbidity, tumor stage, ablation modality, procedural approach, and operator experience. Third, differences in complication definitions, severity classifications, ascertainment windows, and follow-up duration may have introduced outcome-measurement bias and limited comparability across studies. Fourth, publication bias was assessed only for R.E.N.A.L. outcomes with a sufficient number of studies, and funnel-plot asymmetry suggested that publication bias or small-study effects could not be excluded. Publication bias could not be meaningfully evaluated for PADUA, (MC)^2^, or individual risk-factor analyses because too few studies were available. Finally, because no direct comparative meta-analysis was possible and predictive-performance measures were inconsistently reported, the relative performance of the three scoring systems remains uncertain.

Overall, the findings suggest that higher R.E.N.A.L., PADUA, and (MC)^2^ score categories may identify patients with greater odds of complications after renal ablation, particularly major complications. However, the evidence is limited by retrospective study designs, sparse data for several outcomes, clinical and methodological heterogeneity, inconsistent complication reporting, and possible publication bias. Future multicenter prospective studies should directly compare the scoring systems, use standardized definitions and follow-up periods for complications, adjust for relevant patient-, tumor-, and procedure-related confounders, and report discrimination, calibration, and clinical utility before any scoring system can be recommended as a definitive predictive model.

## 7 Conclusion

As easy scoring methods, the R.E.N.A.L., PADUA, and (MC)2 scoring systems can predict complications of renal ablation, especially major complications. Subgroup analysis shows that tumor diameter, distance from the adjacent collecting system, central tumor location, MI history, and complicated DM history are associated with increased complications.
