# Supplementary material for: Can risk scoring systems (R.E.N.A.L, PADUA and (MC)2 scoring systems) and related risk factors predict complications of renal ablation? a meta-analysis
Source: Front Oncol. 2026 Aug 21;16:1753281. doi: 10.3389/fonc.2026.1753281 (PMC13541456; doi:10.3389/fonc.2026.1753281)
Supplement: Supplementary file 1 [file Table1.docx]

| PubMed | (("renal tumor"[Title/Abstract] OR "renal tumors"[Title/Abstract] OR "kidney tumor"[Title/Abstract] OR "kidney tumors"[Title/Abstract] OR "renal mass"[Title/Abstract] OR "renal masses"[Title/Abstract] OR "renal cell carcinoma"[Title/Abstract] OR RCC[Title/Abstract] OR "Kidney Neoplasms"[Mesh]) AND (ablation[Title/Abstract] OR ablat*[Title/Abstract] OR cryoablation[Title/Abstract] OR cryoablat*[Title/Abstract] OR "radiofrequency ablation"[Title/Abstract] OR "microwave ablation"[Title/Abstract] OR "thermal ablation"[Title/Abstract] OR "Ablation Techniques"[Mesh]) AND ("R.E.N.A.L."[Title/Abstract] OR "RENAL nephrometry"[Title/Abstract] OR nephrometry[Title/Abstract] OR PADUA[Title/Abstract] OR "(MC)2"[Title/Abstract] OR "MC2"[Title/Abstract] OR "scoring system"[Title/Abstract] OR "risk score"[Title/Abstract]) AND (complication*[Title/Abstract] OR "adverse event"[Title/Abstract] OR "adverse events"[Title/Abstract] OR morbidity[Title/Abstract] OR outcome*[Title/Abstract])) |
| --- | --- |

| Embase | ('kidney tumor'/exp OR 'renal cell carcinoma'/exp OR 'renal tumor*':ti,ab OR 'kidney tumor*':ti,ab OR 'renal mass*':ti,ab OR 'renal cell carcinoma':ti,ab OR rcc:ti,ab) AND ('ablation therapy'/exp OR 'radiofrequency ablation'/exp OR 'cryoablation'/exp OR 'microwave ablation'/exp OR ablat*:ti,ab OR cryoablat*:ti,ab OR 'radiofrequency ablation':ti,ab OR 'microwave ablation':ti,ab OR 'thermal ablation':ti,ab) AND ('r.e.n.a.l.':ti,ab OR 'renal nephrometry':ti,ab OR nephrometry:ti,ab OR padua:ti,ab OR '(mc)2':ti,ab OR mc2:ti,ab OR 'scoring system':ti,ab OR 'risk score':ti,ab) AND ('complication'/exp OR complication*:ti,ab OR 'adverse event*':ti,ab OR morbidity:ti,ab OR outcome*:ti,ab) |
| --- | --- |

| Cochrane Library | ("renal tumor" OR "renal tumors" OR "kidney tumor" OR "kidney tumors" OR "renal mass" OR "renal masses" OR "renal cell carcinoma" OR RCC):ti,ab,kw AND (ablation OR ablat* OR cryoablation OR cryoablat* OR "radiofrequency ablation" OR "microwave ablation" OR "thermal ablation"):ti,ab,kw AND ("R.E.N.A.L." OR "RENAL nephrometry" OR nephrometry OR PADUA OR "(MC)2" OR MC2 OR "scoring system" OR "risk score"):ti,ab,kw AND (complication* OR "adverse event*" OR morbidity OR outcome*):ti,ab,kw |
| --- | --- |

| Web of Science | TS=(("renal tumor*" OR "kidney tumor*" OR "renal mass*" OR "renal cell carcinoma" OR RCC) AND (ablation OR ablat* OR cryoablation OR cryoablat* OR "radiofrequency ablation" OR "microwave ablation" OR "thermal ablation") AND ("R.E.N.A.L." OR "RENAL nephrometry" OR nephrometry OR PADUA OR "(MC)2" OR MC2 OR "scoring system" OR "risk score") AND (complication* OR "adverse event*" OR morbidity OR outcome*)) |
| --- | --- |
